# Safety and Feasibility of a Simplified Perioperative Hemodialysis Protocol in Hemodialysis Patients Undergoing Off-Pump Coronary Artery Bypass Grafting

**DOI:** 10.5761/atcs.oa.25-00220

**Published:** 2026-07-16

**Authors:** Toshio Doi, Saori Nagura, Kanetsugu Nagao, Masaya Aoki, Shigeki Yokoyama, Shigeyuki Yamashita, Kazuaki Fukahara, Naoki Yoshimura

**Affiliations:** 1 First Department of Surgery, University of Toyama, Toyama, Toyama, Japan; 2 Department of Surgery, Imizu Municipal Hospital, Toyama, Toyama, Japan

**Keywords:** end-stage renal disease, perioperative hemodialysis management, off-pump coronary artery bypass grafting

## Abstract

**Purpose:**

Patients on hemodialysis (HD) undergoing coronary artery bypass grafting (CABG) face a high risk of perioperative complications, and the optimal timing of perioperative HD remains unclear. We evaluated the safety and feasibility of a standardized HD protocol in patients undergoing off-pump CABG (OPCAB).

**Methods:**

We retrospectively analyzed 64 HD-dependent patients who underwent elective OPCAB at a single institution between March 2014 and December 2024. The protocol included maintaining a Monday–Wednesday–Friday HD schedule, performing routine HD on Monday morning, conducting OPCAB on Monday afternoon, and resuming HD on postoperative day (POD) 2. Clinical outcomes, unplanned dialysis, and perioperative biochemical changes were assessed.

**Results:**

Seven patients (10.9%) required unplanned renal replacement therapy before the first postoperative HD session for congestive heart failure (n = 3), hyperkalemia (n = 3), or respiratory failure (n = 1). None required additional dialysis after the initial postoperative HD. In-hospital mortality was 3.1%. Postoperative congestive heart failure occurred in 4.7% of patients. Body weight returned to near baseline by POD 7, and serum potassium remained within an acceptable range throughout the perioperative period.

**Conclusion:**

This standardized perioperative HD protocol was feasible for maintenance HD patients undergoing elective OPCAB. However, protocol deviation in 10.9% of patients suggests that further refinement and prospective comparative studies are warranted.

## Introduction

Patients undergoing maintenance hemodialysis (HD) represent one of the highest-risk subgroups among individuals with coronary artery disease; nevertheless, coronary artery bypass grafting (CABG) remains an essential revascularization strategy, particularly for those with multivessel or left main disease.^1–3)^ Compared with non-dialysis patients, dialysis-dependent patients experience substantially higher perioperative risk, including hemodynamic instability, stroke, bleeding, infection, prolonged hospitalization, and mortality.^4–6)^

A major challenge in managing these patients is their complete loss of renal fluid and electrolyte regulation, which predisposes them to volume overload, hyperkalemia, and metabolic abnormalities.^7)^ Furthermore, most institutions rely on individualized dialysis scheduling, and no standardized guidance exists regarding the optimal timing of preoperative or postoperative dialysis.^8–10)^ Early postoperative dialysis may precipitate bleeding or hypotension, whereas delayed dialysis increases the risk of heart failure exacerbation or severe hyperkalemia.

To address these challenges, our institution adopted a simple, unified perioperative dialysis protocol for HD patients undergoing CABG that required no additional or unscheduled dialysis sessions either before or after surgery. This study aimed to evaluate the safety and feasibility of this simple, reproducible dialysis-scheduling strategy in dialysis-dependent patients undergoing CABG, focusing particularly on the early postoperative clinical course and the stability of biochemical parameters.

## Materials and Methods

### Study design and patient population

This single-center retrospective observational study was conducted at the University of Toyama Hospital. Among 734 consecutive patients who underwent isolated CABG between March 2014 and December 2024, 71 patients (9.7%) were receiving maintenance HD preoperatively. Patients requiring cardiopulmonary bypass, emergency procedures, or intraoperative conversion to on-pump CABG were excluded. A total of 64 patients met the inclusion criteria. All operations were performed using standardized off-pump coronary artery bypass (OPCAB) techniques by 5 board-certified cardiovascular surgeons.

The study protocol was approved by the Institutional Review Board of the University of Toyama Hospital (Approval No. R2020065). The requirement for individual informed consent was waived owing to the retrospective nature of the study.

### Preoperative HD management

All patients were admitted 1 week prior to surgery, and HD was standardized to a Monday–Wednesday–Friday schedule. Preoperative optimization included confirmation of dry weight, adjustment of ultrafiltration targets to avoid intradialytic hypotension, and stabilization of serum potassium and acid–base status. On the day of surgery (Monday), all patients underwent routine morning HD. The ultrafiltration volume during morning HD was determined based on the patient’s usual dry weight, pre-dialysis body weight, blood pressure, clinical symptoms, and intradialytic tolerance. The target body weight was set as close as possible to the usual dry weight, while avoiding excessive ultrafiltration that could cause intravascular volume depletion or hemodynamic instability during anesthesia induction. Body weight measured after morning HD was defined as the preoperative body weight for subsequent perioperative comparisons. After confirming hemodynamic stability, OPCAB was performed in the afternoon.

### Surgical technique and perioperative management

All procedures were performed via median sternotomy under general anesthesia. Arterial grafts included the left internal thoracic artery (ITA) and, when indicated, the right ITA. Saphenous vein grafts (SVGs) were harvested and used according to the target vessel anatomy. The ITA was harvested using either the skeletonized or pedicled technique at the surgeon’s discretion. Proximal anastomoses of SVGs were constructed using a clampless proximal anastomosis device to avoid aortic clamping.

In patients at high risk for perioperative hemodynamic instability—including those with severe left main disease (≥90% stenosis) or severe left ventricular dysfunction (left ventricular ejection fraction [LVEF] <35%)—prophylactic mechanical circulatory support was employed. An intra-aortic balloon pump (IABP) was inserted before induction of general anesthesia and maintained intraoperatively. In patients with severe ventricular dysfunction in whom IABP support was expected to be insufficient, prophylactic Impella (Abiomed, Danvers, MA, USA) placement was considered preoperatively.

During surgery, crystalloid administration was restricted to minimize volume overload. Hemodynamic stability was maintained using head-down positioning when necessary and proactive use of norepinephrine to achieve a target mean arterial pressure of 50–60 mmHg. Systemic heparinization was performed after graft harvesting, targeting an activated clotting time (ACT) of 300 s; protamine was administered after completion of distal and proximal anastomoses. Shed blood was recovered intraoperatively using the Cell Saver Elite system (Haemonetics, Braintree, MA, USA) and autotransfused as appropriate.

Postoperatively, all patients were managed in the intensive care unit (ICU) for 2 days. Basal intravenous fluid administration was standardized at 1000 mL/day, with adjustments made based on clinical indications. During the early postoperative period, systolic blood pressure was generally maintained between 80 and 120 mmHg under continuous hemodynamic monitoring while ensuring adequate end-organ perfusion. Blood pressure targets were individualized, with relatively higher targets applied in patients with significant carotid artery stenosis or cerebrovascular disease. After confirming hemostatic stability at 24 h, low-dose continuous intravenous heparin infusion (8000 U/day) was initiated. Arterial blood gas analysis was performed every 6 h, and serum potassium was maintained within the target range of 4.0–5.5 mmol/L using standard therapies such as bicarbonate infusion or glucose–insulin treatment when necessary. Oral antiplatelet therapy and enteral intake were initiated on postoperative day (POD) 2, at which time intravenous fluids were gradually reduced. Cefazolin (1 g/day) was administered for 4 postoperative days following the institutional infection control protocol.

### Postoperative HD management

On POD 2 (Wednesday), patients were transferred from the ICU to the dialysis unit, where HD was resumed according to the predetermined Monday–Wednesday–Friday schedule. For the first postoperative HD session, ultrafiltration volume was individualized based on a comprehensive assessment of total body fluid accumulation and intravascular volume status. In addition to postoperative body weight gain, blood pressure trends, vasopressor requirements, peripheral edema, pulmonary congestion on chest radiography, oxygenation status, and laboratory findings were considered. When intravascular hypovolemia was suspected despite postoperative weight gain, ultrafiltration was reduced or withheld to avoid dialysis-induced hypotension. Dialysis duration was generally maintained according to the usual preoperative session length but adjusted according to hemodynamic tolerance. Ultrafiltration goals were titrated daily with the explicit aim of returning to the preoperative dry weight within 1 week. To minimize bleeding risk, nafamostat mesylate (30 mg/h) was used during HD sessions for approximately 1 week after surgery. Unscheduled HD or continuous renal replacement therapy (CRRT) was initiated for refractory hyperkalemia (serum K+ >6.0 mmol/L despite medical therapy with bicarbonate, glucose–insulin, or potassium binders), symptomatic volume overload with pulmonary edema or respiratory distress, severe metabolic acidosis (pH <7.20 or HCO3- <15 mEq/L) unresponsive to intravenous bicarbonate, or uremic manifestations. Each case requiring additional dialysis was reviewed jointly by the cardiovascular surgeon and nephrologist. These criteria were applied consistently throughout the study period. Ultrafiltration targets for the first postoperative dialysis session were determined according to clinical assessment of volume status, including physical examination, body weight relative to preoperative dry weight, chest radiography, and hemodynamic parameters. The initial ultrafiltration target was typically set at approximately 50% of weight gain from dry weight, with real-time adjustment according to intradialytic blood pressure response and patient tolerance. Standard dialysis duration was 4 h, with extension when necessary in patients requiring greater fluid removal or exhibiting hemodynamic instability.

### Data collection and outcomes

Data were extracted from electronic medical records, dialysis charts, laboratory databases, and operative/anesthesia reports.

The primary outcome was the requirement for unplanned renal replacement therapy, defined as any HD or CRRT performed outside the prescribed Monday–Wednesday–Friday schedule or for emergent clinical indications. Administrative rescheduling without treatment was not considered an event. Secondary outcomes included in-hospital mortality, postoperative stroke, mediastinitis, pneumonia, duration of mechanical ventilation, ICU length of stay, and postoperative length of hospitalization.

### Statistical analysis

Continuous variables were expressed as median (interquartile range [IQR]). Categorical variables were presented as counts and percentages. Patients were stratified according to the presence or absence of unplanned postoperative HD. Perioperative variables were compared using the Mann–Whitney U test for continuous variables and Fisher’s exact test for categorical variables. Serial biochemical measurements were compared between baseline (preoperative) and each postoperative time point using the Wilcoxon signed-rank test. A two-sided p-value <0.05 was considered statistically significant. Statistical analyses were performed using JMP 18 Statistical Software (SAS Institute Inc., Cary, NC, USA).

## Results

### Patient characteristics

During the study period, 71 patients undergoing maintenance HD received isolated OPCAB. After excluding 2 patients who required cardiopulmonary bypass and 5 patients who underwent emergency procedures, 64 patients were included in the final analysis. Preoperative patient characteristics are summarized in **Table 1**. The median age was 69.5 years (IQR 63.0–73.0), and 55 patients (85.9%) were male. Hypertension, dyslipidemia, and diabetes mellitus were present in 50 (78.1%), 19 (29.7%), and 52 (81.2%) patients, respectively. Chronic atrial fibrillation was observed in 5 patients (7.8%), cerebrovascular disease in 10 (15.6%), peripheral artery disease in 19 (29.7%), and prior myocardial infarction in 36 (56.3%). The median number of diseased coronary vessels was 3.0 (IQR 2.0–3.0), and 20 patients (31.2%) had left main disease. Median LVEF was 53.5% (IQR 43.0–62.7), and 11 patients (17.2%) had severe LV dysfunction (LVEF ≤35%). Diabetic nephropathy was the most common etiology of end-stage renal disease (51 patients, 79.7%). Median duration of HD was 4.0 years (IQR 1.0–9.7). Median preoperative blood urea nitrogen (BUN), creatinine, potassium, and hemoglobin levels were 42.0 mg/dL (IQR 36.1–51.9), 7.7 mg/dL (IQR 6.2–9.5), 4.5 mmol/L (IQR 4.1–5.1), and 10.9 g/dL (IQR 10.1–12.0), respectively. Median ultrafiltration volume during planned preoperative HD on the day of surgery was 450.0 mL (IQR 1.5–1350.0). No significant differences in preoperative characteristics were observed between the unplanned and non-unplanned HD groups.

**Table 1:** Preoperative characteristics of patients

Variable	Total (n = 64)	Unplanned HD(+) (n = 7)	Unplanned HD(−) (n = 57)	p Value
Age (years)	69.5 (63.0–73.0)	65.0 (61.0–75.0)	70.0 (63.0–73.0)	0.682
Male	55 (85.9)	6 (85.7)	49 (85.9)	1
Hypertension	50 (78.1)	6 (85.7)	44 (77.2)	1
Dyslipidemia	19 (29.7)	1 (14.3)	18 (31.6)	0.663
Diabetes mellitus	52 (81.2)	7 (100)	45 (79.0)	0.33
History of smoking	41 (64.1)	4 (57.1)	37 (64.9)	0.694
Effort angina pectoris	28 (43.7)	1 (14.3)	27 (47.4)	0.125
Old myocardial infarction	36 (56.3)	6 (85.7)	30 (52.6)	0.125
Number of coronary stenosis	3.0 (2.0–3.0)	3.0 (2.0–3.0)	3.0 (2.0–3.0)	0.862
Left main trunk stenosis	20 (31.2)	1 (14.3)	19 (33.3)	0.418
Left ventricular ejection fraction (%)	53.5 (43–62.7)	43.0 (28.0–57.0)	55.0 (43.5–63.0)	0.134
Left ventricular dysfunction (<35%)	11 (17.2)	2 (28.6)	9 (15.8)	0.593
Previous stroke	10 (15.6)	0 (0)	10 (17.5)	0.584
Chronic atrial fibrillation	5 (7.8)	1 (14.3)	4 (7.0)	0.45
Chronic obstructive pulmonary disease	1 (1.6)	0 (0)	1 (1.6)	1
Peripheral arterial disease	19 (29.7)	3 (42.9)	16 (28.1)	0.414
Duration of HD	4.0 (1.0–9.7)	7.0 (5.0–11.0)	3.0 (1.0–9.0)	0.083
Cause of HD				
Diabetic	51 (79.7)	7 (100)	44 (77.2)	0.328
Chronic glomerulonephritis	4 (6.2)	0 (0)	4 (7.0)	1
Nephrosclerosis	4 (6.2)	0 (0)	4 (7.0)	1
IgA nephropathy	2 (3.1)	0 (0)	2 (3.5)	1
Polycystic kidney disease	2 (3.1)	0 (0)	2 (3.5)	1
Renal tuberculosis	1 (1.6)	0 (0)	1 (1.8)	1
Preoperative laboratory data				
Blood urea nitrogen (mg/dL)	42.0 (36.1–51.9)	38.0 (28.6–44.0)	42.7 (36.75–52.85)	0.143
Creatinine (mg/dL)	7.7 (6.2–9.5)	7.8 (5.4–12.6)	7.6 (6.2–9.4)	0.659
Potassium (mmol/L)	4.5 (4.1–5.1)	4.3 (3.9–5.3)	4.5 (4.1–5.1)	0.948
Hemoglobin (g/dL)	10.9 (10.1–12.0)	10.7 (10.4–11.4)	11.0 (10.0–12.2)	0.838
Fluid removal on the day of operation (mL)	450.0 (1.5–1350.0)	1.6 (0.7–1550.0)	550.0 (1.5–1350.0)	0.338

Data are presented as median (IQR) or number of patients (%).

HD, hemodialysis

### Operative findings

Operative details are shown in **Table 2**. No patient developed hemodynamic instability during the induction of general anesthesia despite undergoing HD immediately before surgery. The median number of bypass grafts was 3.0 (IQR 2.0–4.0). The ITA was used in 59 patients (92.2%), and SVGs in 62 patients (96.8%). The aorta no-touch technique was used in 3 patients (4.7%), and complete revascularization was achieved in 50 patients (78.1%). No patient required unplanned conversion to cardiopulmonary bypass. Median operative time was 221.5 min (IQR 186.2–260.5). Median intraoperative infusion volume and blood loss were 2815.0 mL (IQR 2142.5–3482.5) and 970.0 mL (IQR 690.0–1745.0), respectively. Blood transfusion was required in 43 patients (67.2%). Intraoperative findings were similar between the 2 groups.

**Table 2:** Intraoperative data

Variable	Total (n = 64)	Unplanned HD(+) (n = 7)	Unplanned HD(−) (n = 57)	p Value
Operation time (min)	221.5 (186.2–260.5)	215.0 (198.0–255.0)	222 (185.5–263.5)	0.738
Number of distal anastomoses	3.0 (2.0–4.0)	2.0 (2.0–4.0)	3.0 (2.0–4.0)	0.588
Preoperative IABP support	14 (21.8)	2 (28.6)	12 (21.1)	0.641
Preoperative Impella support	1 (1.6)	1 (14.2)	0 (0)	1
Amount of fluid intake (mL)	2815.0 (2142.5–3482.5)	3230.0 (2620.0–3780.0)	2800 (2132.5–3450.0)	0.216
Bleeding amount (mL)	970.0 (690.0–1745.0)	1135.0 (530.0–1530.0)	930 (690.0–1385.0)	0.561
Transfusion	43 (67.2)	4 (57.1)	39 (68.4)	0.674
Conversion to on-pump CABG	0 (0)	0 (0)	0 (0)	NA
Use of ITA	59 (92.2)	6 (85.7)	53 (93.0)	0.45
Use of SVG	62 (96.8)	7 (100)	55 (96.5)	1
Aorta no-touch	3 (4.7)	0 (0)	3 (5.3)	1
Complete revascularization	50 (78.1)	5 (71.4)	45 (79.0)	0.641

Data are presented as median (IQR) or number of patients (%).

CABG, coronary artery bypass grafting; HD, hemodialysis; IABP, intra-aortic balloon pumping; ITA, internal thoracic artery; NA, not applicable; SVG, saphenous vein graft

### Postoperative course

Postoperative outcomes are summarized in **Table 3**. In-hospital mortality occurred in 2 patients (3.1%), due to cardiac arrest following acute limb ischemia and severe pneumonia, respectively. Major postoperative complications included stroke in 1 patient (1.6%), pneumonia in 4 patients (6.3%), and mediastinitis in 1 patient (1.6%). Postoperative atrial fibrillation occurred in 21 patients (32.8%). The median duration of mechanical ventilation was 11.0 h (IQR 10.0–12.0), and only 1 patient (1.6%) required prolonged ventilation (>72 h). The median ICU stay and postoperative hospital stay were 2.0 days (IQR 2.0–2.0) and 21.5 days (IQR 18.8–29.0), respectively. In the unplanned HD group, in-hospital mortality and the incidence of heart failure were significantly higher, and both ICU and postoperative hospital stays were significantly longer. Major Adverse Cardiac and Cerebrovascular Events (MACCE) occurred in 3 patients, comprising 5 events, including 3 cases of heart failure and 2 cases of cerebral infarction. Because the number of MACCE events was small (n = 5), statistical analyses for associated factors were not performed.

**Table 3:** Postoperative outcomes

Variable	Total (n = 64)	Unplanned HD(+) (n = 7)	Unplanned HD(−) (n = 57)	p Value
30-day mortality	0 (0)	0 (0)	0 (0)	NA
In-hospital mortality	2 (3.1)	2 (28.6)	0 (0)	0.01
ICU stay (days)	2 (2–2)	4.0 (2.0–4.0)	2.0 (2.0–2.0)	<0.001
Postoperative hospital stay (days)	21.5 (18.8–29.0)	61.0 (19.0–106.0)	21.0 (18.0–26.5)	0.022
Re-exploration for bleeding	0 (0)	0 (0)	0 (0)	NA
Permanent neurological deficit	1 (1.6)	0 (0)	1 (1.8)	1
Myocardial infarction	0 (0)	0 (0)	0 (0)	NA
Congestive heart failure	3 (4.7)	3 (42.9)	0 (0)	<0.001
New onset atrial fibrillation	21 (32.8)	4 (57.1)	17 (29.5)	0.204
Life-threatening arrhythmia	1 (1.6)	1 (14.3)	0 (0)	0.109
Mechanical ventilation (h)	11.0 (10.0–12.0)	12.0 (11.0–14.0)	11.0 (10.0–12.0)	0.112
Prolonged ventilation (>72 h)	1 (1.6)	1 (14.3)	0 (0)	0.109
Tracheostomy	1 (1.6)	1 (14.3)	0 (0)	0.109
Pneumonia	4 (6.3)	2 (28.6)	2 (3.5)	0.055
Mediastinitis	1 (1.6)	0 (0)	1 (1.8)	1
Wound infection	5 (7.8)	1 (14.3)	4 (7.0)	0.45
Visceral ischemia	0 (0)	0 (0)	0 (0)	NA

Data are presented as median (IQR) or number of patients (%).

HD, hemodialysis; NA, not applicable; ICU, intensive care unit

**Fig. 1:**
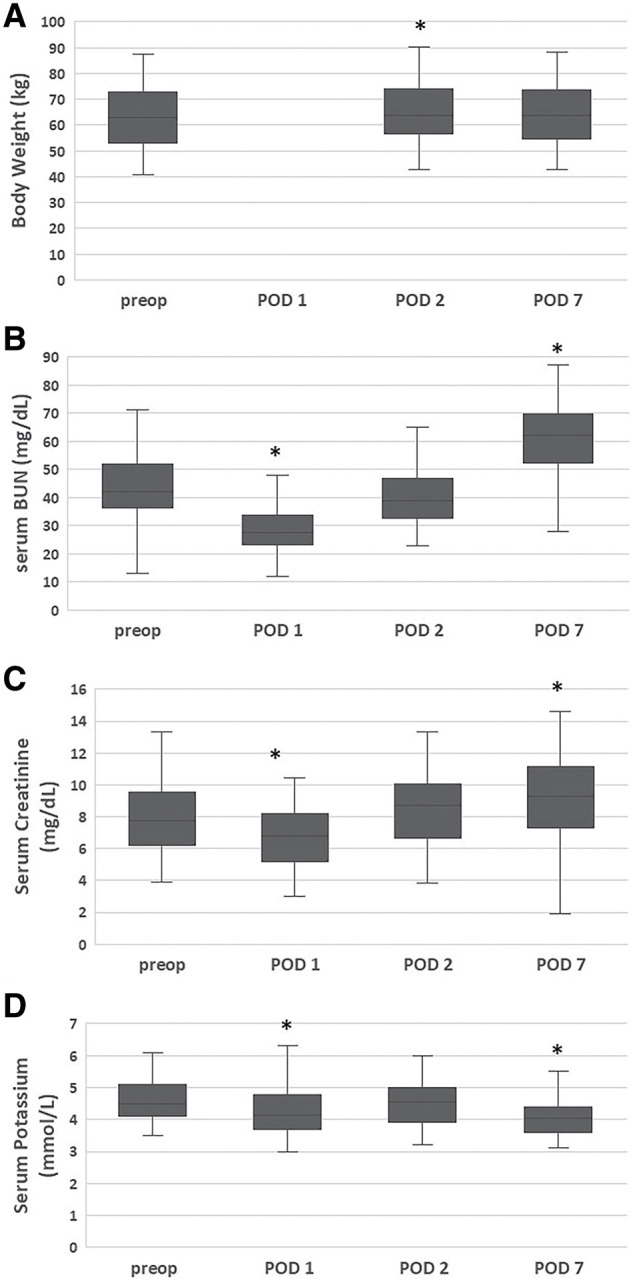
Perioperative trends in body weight and biochemical parameters after OPCAB. (**A**) Body weight: Body weight increased slightly on postoperative day 2 (POD 2) but returned close to preoperative levels by POD 7. (**B**) BUN: BUN decreased on POD 1 following surgery and dialysis withdrawal, increased again by POD 2, and gradually increased by POD 7, consistent with the regular 48-h HD interval. (**C**) Serum creatinine: Creatinine levels showed minimal fluctuation during the early postoperative period and demonstrated interpatient variability by POD 7. (**D**) Serum potassium: Potassium levels remained within a clinically acceptable range throughout the postoperative period, indicating stable electrolyte control under the standardized dialysis schedule. Data are presented as median (interquartile range). Asterisks (*) indicate statistically significant differences compared with preoperative values (p <0.05). OPCAB, off-pump coronary artery bypass grafting; BUN, blood urea nitrogen; HD, hemodialysis

Postoperative dialysis-related findings are summarized in **Table 4**. Seven patients (10.9%) required unplanned renal replacement therapy before the first scheduled postoperative HD session on POD 2. Indications included congestive heart failure (n = 3), hyperkalemia (n = 3), and respiratory failure (n = 1). No patient required additional dialysis between scheduled sessions after the initial postoperative dialysis. The median ultrafiltration volume during the first postoperative HD session was 1500.0 mL (IQR 2.3–2170.0). Median body weight increased from 62.7 kg (IQR 53.2–72.7) preoperatively (after the morning HD session on the day of surgery) to 63.9 kg (IQR 56.7–74.0) on POD 2 (p <0.05), but returned to near-baseline values by POD 7 at 63.8 kg (IQR 54.6–73.8) (**Fig. 1A**).

**Table 4:** Postoperative HD management

Variable	Total (n = 64)
Unplanned HD	7 (10.9)
Reason for unplanned HD	
Congestive heart failure	3 (4.7)
Respiratory failure	1 (1.6)
Hyperkalemia	3 (4.7)
Fluid removal on the first dialysis after operation (mL)	1500.0 (2.3–2170.0)

Data are presented as median (IQR) or number of patients (%).

HD, hemodialysis

### Biochemical parameters

Perioperative biochemical trends are illustrated in **Figure 1**. Serum BUN decreased from 42.0 mg/dL (IQR 36.1–51.9) preoperatively to 27.6 mg/dL (IQR 23.3–33.8) on POD 1, increased to 39.0 mg/dL (IQR 32.7–47.0) on POD 2, and further increased to 62.0 mg/dL (IQR 37.0–69.8) by POD 7 (**Fig. 1B**). Serum creatinine demonstrated a similar trend, measuring 7.7 mg/dL (IQR 6.2–9.5) preoperatively, 6.8 mg/dL (IQR 5.1–8.1) on POD 1, 8.7 mg/dL (IQR 6.6–10.0) on POD 2, and 9.2 mg/dL (IQR 7.3–11.1) on POD 7 (**Fig. 1C**). Serum potassium levels remained within a clinically acceptable range throughout the perioperative period, changing from 4.5 mmol/L (IQR 4.1–5.1) preoperatively to 4.1 mmol/L (IQR 3.7–4.7) on POD 1, 4.5 mmol/L (IQR 3.9–5.0) on POD 2, and 4.0 mmol/L (IQR 3.6–4.4) on POD 7 (**Fig. 1D**).

## Discussion

Perioperative management in HD patients is inherently challenging. These patients are predisposed to fluid overload–related heart failure and respiratory compromise, coagulopathy-associated bleeding, hyperkalemia, and uremic complications.^11,12)^ Because they lack intrinsic renal regulatory function for water and electrolyte homeostasis, even minimal shifts in fluid or electrolyte balance can precipitate hemodynamic collapse or life-threatening arrhythmias.^13,14)^ Moreover, after CABG or other cardiac surgeries, the combined effects of surgical stress, inflammation, transfusion, and fluid administration further predispose HD patients to postoperative complications. Consequently, postoperative morbidity and mortality rates in HD patients are consistently higher than in non-HD patients.^4–6,15)^ From this perspective, overall perioperative outcomes are influenced not only by surgical technique but also by the design and timing of the dialysis schedule throughout the perioperative period. The low rates of unplanned dialysis and in-hospital mortality observed in this study support the clinical value of a standardized schedule for reducing postoperative risk.

Several issues must be considered when determining perioperative HD strategies, including the timing of preoperative HD, the modality and initiation of postoperative renal replacement therapy, and anticoagulation management. Despite numerous reports, consensus remains lacking regarding the optimal approach.^16,17)^ Regarding preoperative HD, several studies have suggested that HD on the day before surgery is safer in cardiac surgical patients.^11,18,19)^ This is based on concerns that same-day HD may cause intravascular volume depletion or rapid osmotic shifts, increasing the risks of intraoperative hypotension, hemodynamic instability, and bleeding related to anticoagulant use during dialysis. In our cohort, intraoperative hemodynamics during anesthesia induction were stable in all cases, supporting the safety of same-day HD. However, intraoperative blood loss and transfusion requirements tended to be higher, suggesting that anticoagulation used during HD may have contributed to increased bleeding. Future optimization of anticoagulation strategies—such as dose reduction or heparin-free HD—should therefore be explored.

A key feature of our protocol was the use of a calendar-based fixed schedule rather than a reactive approach based primarily on biochemical parameters. This strategy aimed to balance several competing priorities: (1) minimizing bleeding risk by avoiding dialysis during the first 48 postoperative hours when hemostasis is most vulnerable; (2) preventing severe metabolic derangements through timely dialysis resumption; and (3) facilitating operational efficiency through predictable scheduling. However, this fixed-schedule approach has inherent limitations. Seven patients (10.9%) required unplanned dialysis despite the standardized protocol, suggesting that a uniform 48-h interval may not be optimal for all patients. The indications for unplanned dialysis—congestive heart failure (n = 3), hyperkalemia (n = 3), and respiratory failure (n = 1)—indicate that certain patients may benefit from earlier dialysis resumption or closer perioperative monitoring. Preoperative volume status, intraoperative fluid balance, and left ventricular function may influence individual tolerance to delayed dialysis.

We attempted to identify predictors of unplanned dialysis by comparing baseline and operative characteristics between patients who did and did not require unscheduled dialysis. However, the small number of events (n = 7) precluded meaningful statistical analysis or reliable risk stratification. Multivariate analysis was not feasible because the low event-to-variable ratio would likely produce overfitting and unreliable estimates. Therefore, the patient subgroups at greatest risk for requiring unplanned dialysis under this protocol could not be clearly identified. Future studies with larger cohorts are needed to establish a risk stratification model to identify patients who may benefit from modified dialysis timing, such as dialysis initiation on POD 1 or more frequent biochemical monitoring during the 48-h interval. Such models may incorporate preoperative factors (LVEF, residual renal function, volume status), intraoperative variables (blood loss, transfusion requirement, fluid balance), and early postoperative markers.

Fluid removal during the first postoperative dialysis session represents a particular clinical challenge. Despite weight gain averaging 2.1 kg on POD 2, some patients exhibited relative intravascular depletion because of postoperative third-spacing associated with surgical trauma and inflammation. The median ultrafiltration volume during the first dialysis session was 1500 mL, which was lower than the total weight gain in most patients, reflecting a cautious approach to avoid hypotension and hemodynamic compromise. The wide variation in ultrafiltration volume (0–2860 mL) was partly attributable to the inclusion of 5 patients in whom ultrafiltration was withheld to preserve hemodynamic stability, highlighting the need for individualized volume management beyond rigid protocol-based targets. The ultrafiltration strategy was adjusted according to clinical assessment and real-time hemodynamic monitoring to balance adequate fluid removal against the risk of intravascular depletion. The gradual return to preoperative dry weight, with body weight approaching baseline by POD 7, suggests that this conservative initial approach was appropriate in most patients. However, incorporation of objective volume assessment methods—such as bioimpedance analysis or echocardiographic evaluation—may further improve the safety and consistency of postoperative fluid management in future protocols.

The temporal changes in body weight and biochemical parameters further support the physiological appropriateness of this standardized schedule. Body weight increased transiently on POD 2 but returned to near-baseline levels by POD 7, indicating that postoperative fluid accumulation was appropriately corrected through scheduled HD. BUN and creatinine fluctuated within ranges predictable for a 48-h interdialytic interval, and no excessive solute accumulation was observed. Serum potassium remained within the safe range throughout, minimizing the risk of life-threatening arrhythmias. These findings confirm that delaying the first postoperative HD session until POD 2 did not lead to adverse metabolic consequences.

Standardizing the perioperative dialysis schedule also carries operational advantages. A predictable schedule facilitates communication between the operating room, dialysis unit, and ICU. This approach facilitates securing dialysis beds, organizing staff assignments, minimizing scheduling conflicts, and improving infection control measures. Particularly in regional hospitals with limited resources, eliminating the need for weekend dialysis by selecting Monday as the operative day not only reduces staff workload but also enhances patient safety. Previous studies have demonstrated that perioperative care pathways improve interdisciplinary coordination, reduce hospital stay, and optimize resource utilization.^20,21)^ The standardized HD schedule evaluated in this study may function as an extension of such pathways and serve as a practical educational tool for training junior physicians and nurses.

This study has several limitations. First, it was a retrospective single-center analysis without a control group using alternative HD schedules; therefore, the superiority of this protocol cannot be directly compared with that of other strategies. Second, the study focused on short-term postoperative outcomes, and its influence on long-term survival or cardiovascular events remains undetermined. Third, the present cohort consisted exclusively of elective OPCAB cases, and patients requiring emergency surgery, cardiopulmonary bypass, or intraoperative conversion were excluded. Therefore, the study population likely represented a relatively stable subgroup of HD-dependent patients. The applicability of this protocol to more complex or hemodynamically unstable patients, including those with cardiogenic shock, acute coronary syndrome, or severe circulatory compromise, remains uncertain. In such settings, individualized renal replacement strategies or earlier initiation of continuous hemodiafiltration may be more appropriate. Finally, the small number of patients requiring unplanned dialysis (n = 7) precluded identification of factors associated with protocol deviation. Consequently, risk stratification criteria could not be established, and patient subgroups that might benefit from alternative dialysis timing could not be clearly defined. Larger multicenter studies are therefore needed to identify predictors of unplanned dialysis requirement and to establish evidence-based criteria for individualizing dialysis timing within a standardized perioperative framework.

## Conclusion

Our standardized perioperative HD protocol was feasible in HD-dependent patients undergoing elective OPCAB. However, 10.9% of patients required deviation from the planned dialysis schedule, and 4.7% developed postoperative congestive heart failure, indicating the need for further protocol refinement. Because this study lacked a control group, the present protocol should be interpreted as a feasible perioperative management strategy for selected stable patients rather than an optimal approach. Prospective comparative studies are warranted to validate its clinical utility.

## Declarations

### Data availability

The data that support the findings of this study are available from the corresponding author upon reasonable request. The data are not publicly available due to privacy or ethical restrictions.
